# Supplementary material for: Are carbon emissions trading and green financial instruments synergistic? -Comprehensive quantitative research based on content analysis
Source: PLoS One. 2024 Mar 7;19(3):e0298601. doi: 10.1371/journal.pone.0298601 (PMC10919601; doi:10.1371/journal.pone.0298601)
Supplement: S1 Data — (ZIP) [file pone.0298601.s001.zip › Data/地级市Z_F工作报告.zip!/║╙─╧/╨┼╤⌠╩╨/└┤╫╘╣1⁄2╓┌║┼í╛┬φ┐╦╩2╛▌═°í┐_2013.docx]

各位代表：

　　现在，我代表信阳市人民政府，向大会作工作报告，请予审议，并请各位政协委员和其他列席的同志提出意见。

　　一、2012年工作回顾

　　2012年是我市持续求进、务实发展、积极作为的一年。在省委、省政府和市委的正确领导下，我们深入贯彻落实科学发展观，坚持“四个重在”实践要领，紧紧依靠全市人民，全面落实市四次党代会部署，勇当中原经济区建设前锋，较好地完成了市四届人大一次会议确定的各项目标任务，全市经济社会发展持续保持好的趋势、好的态势和好的气势。

　　一年来，我们持续求进，发展基础进一步夯实。突出围绕主题主线主基调，持续推进“三化”协调发展，全市生产总值突破1400亿元，地方公共财政预算收入突破55亿元，全社会固定资产投资完成1274.5亿元，其中，工业投资完成434亿元，“十大项目”完成投资100.5亿元，激蓝OLED项目被列为省重大科技专项，电子信息等战略支撑产业不断发展壮大，信阳国际家居产业小镇等一批龙头型、基地型、集群型项目在我市开工建设，义乌小商品城等一批商贸物流企业在我市展开战略布局，我市加快发展的基础更加坚实。

　　一年来，我们务实发展，发展载体进一步完善。突出围绕“一个载体、三个体系”建设，加快产业集聚区发展，信阳工业城被批准为省级高新技术产业开发区，正在创建电子信息产业集聚示范区；羊山行政中心商务区开发建设有效推进；信阳作为河南省农村改革发展综合试验区列入《中原经济区规划》，并被中央农村工作领导小组办公室确定为全国农村改革试验联系点；创建国家级潢川经济技术开发区工作得到省委、省政府大力支持；高标准粮田“百千万”建设工程、现代农业产业化集群培育工程等载体建设全面启动，我市加快发展的载体更加完善。

　　一年来，我们积极作为，发展优势进一步提升。突出围绕谋长远、打基础、创优势、增后劲，合力办成了一批影响深远的大事，事关全局的一些重大项目取得了积极进展。淮息·淮固高速公路建成通车，全市所有县城实现20分钟内上高速。京广高铁全线贯通，宁西铁路增建二线电气化改造和京港澳高速公路改扩建工程信阳段开工建设，明港机场前期工程顺利推进，淮河航运基础设施建设取得新的进展，信阳海关加快建设，出山店水库前期工作进展顺利，南湾湖国家重点湖泊生态环境保护试点项目全面启动，设置信阳农林学院获得全国高等学校设置评议委员会通过，我市加快发展的综合优势进一步提升。

　　一年来，我市经济社会发展总体持续、总体提升、总体协调、总体有效。主要体现在：

　　（一）坚持科学发展，经济持续较快增长。认真落实省、市关于促进经济平稳较快增长的若干措施，着力增强运行调节的前瞻性、针对性、有效性，提高政策措施的科学性、连续性、系统性，较好解决了经济运行中的突出矛盾和问题，确保了全市经济平稳较快增长。初步核算，全市生产总值同比增长10.5%，高于全省平均水平；地方公共财政预算收入增长25.1%，位居全省第2位；地方公共财政预算支出278亿元，增长24.8%，高出全省7个百分点；全社会固定资产投资增长21%；社会消费品零售总额增长16%左右；金融机构各项存款余额较年初增长21.3%，贷款余额较年初增长17.3％，均高于全省平均水平。

　　（二）坚持统筹兼顾，“三化”协调持续推进。强化新型城镇化引领。深入实施城乡建设三年大提升行动计划，统筹推进中心城市、县城、中心镇和新型农村社区协调发展，新区开发、老城改造和中心城市组团建设全面加快，浉河三期治理、中心城区内河治理、雨污分流、路网建设、高铁站片区建设等一批重点工程有序实施，中心城市辐射带动能力进一步增强，城乡一体化进程进一步加快，城镇化质量和城市精细化管理水平进一步提升。预计2012年全市城镇化率超过38%，较上年提高近2个百分点。我市再次荣获中国最具幸福感城市，第四次蝉联中国十佳宜居城市。强化新型工业化主导。围绕构建现代产业体系，大力发展高成长性产业、改造提升传统优势产业、培育壮大战略先导产业，以电子信息为主的高端制造和绿色食品、新型建材、纺织服装等主导产业集群集聚态势正在形成。全市工业投资增长60.7%，增速居全省首位；规模以上工业增加值357.4亿元，增长16.5%，高出全省平均水平近2个百分点；规模以上工业实现利润84亿元，增长26.2%，增速居全省首位。节能减排目标圆满完成。强化新型农业现代化基础。我市农田水利基本建设再获省“红旗渠精神杯”。在连续干旱的情况下，我市全年粮食总产量达到117.1亿斤，实现“九连增”。茶叶、粮油、畜禽、水产、花卉等特色农业产业化步伐加快，市级以上农业产业化重点龙头企业发展到298家。全市茶园面积达到210万亩，全年茶叶产量5.2万吨，产值77.2亿元。在人民网主办的城市符号征集活动中，信阳荣居“全国最具影响力十大茶产地”榜首。深入推进农村改革发展综合试验区建设，农村金融不断创新，农村社会保障制度不断完善，农业规模化经营水平和农民组织化程度不断提升，初步形成了多主体经营农业、多元化投资农村的良好态势。

　　（三）坚持重点突破，发展后劲持续提升。坚持把产业集聚区发展、招商引资、项目建设作为战略性举措来抓，以重点突破带动全局发展。突出抓好产业集聚区建设。坚持“三规合一”、“四集一转”、产城融合，不断推动产业集聚区实现更大规模、更高水平、更好效果发展。全市产业集聚区完成固定资产投资突破600亿元，增长39.2%，占全市固定资产投资比重超过50%；产业集聚区实现主营业务收入950.24亿元，占全市规模以上工业的65.1%，较上年提高4.1个百分点，产业集聚区的综合效应和辐射带动作用正在持续显现。突出抓好开放招商。持续推进“四一”招商行动，积极承接产业转移，加强与央企战略合作，开放招商规模不断扩大、质量不断提升、领域不断拓宽。全年实际利用外商直接投资3.5亿美元，增长21.9%；实际到位市外资金210亿元，增长35%，高于全省平均水平；外贸出口总值2.56亿美元，增长44.4%，增速居全省第3位；成功举办第20届信阳国际茶文化节，信阳的知名度和影响力进一步提升。突出抓好项目建设。坚持以项目带动调结构、补短板、增后劲、促发展，强力推进“项目落地年”活动和重大项目攻坚计划，持续强化项目谋划、项目招商、项目落地和项目服务工作机制，严格落实项目联合督查、产业集聚区重点项目攻坚督导和“四集中”、“零阻碍”服务制度，全市107个省市重点项目、149个重大工业转型升级项目扎实推进，共有84个工业项目竣工投产，一批交通、能源、水利等基础设施项目和社会事业项目建成使用，商贸物流、文化旅游、金融保险、邮政、信息服务等行业都依托项目支撑呈现出持续向上的发展趋势。

　　（四）坚持改革创新，发展活力持续增强。突出围绕重点领域和关键环节，持续推进改革创新，为经济社会发展注入了强大动力。国有企业改革不断深化，市直机关所属企业脱钩改制工作全面推进，非公有制经济加快发展；行政管理体制改革扎实推进，事业单位分类改革有序实施，固始直管县改革试点成效明显；医药卫生体制和文化体制改革阶段性重点任务全面完成，集体林权制度改革、农村信用社改革、农电体制改革、县级供销体制改革取得新的成效；财税金融改革稳步推进，“准市政债”一期成功发行10亿元，“人地挂钩”政策获得省批准试点，电力良性发展机制初步建立，资金、土地、电力等瓶颈制约进一步缓解；大力推进自主创新体系建设，新增省级院士工作站2家，新增国家高新技术企业3家。

　　（五）坚持以人为本，人民生活持续改善。省里安排的“十项重点民生工程”和我市“十件实事”基本完成，全市财政用于重点民生的支出211亿元，占全市公共财政支出的75.9％。千方百计增加居民收入。城镇居民人均可支配收入突破17000元，农民人均纯收入突破7000元，分别增长13%和13.9％。大力实施“菜篮子”工程，居民消费价格总水平涨幅控制在3%以内。千方百计扩大就业。全市城镇新增就业人员7.1万人，城镇下岗失业人员再就业2.9万人。千方百计提升保障水平。城镇居民医保和城乡居民基本养老保险制度实现全覆盖，新农合参合率达到98.6%，大病救助试点工作稳步推进，城乡低保和农村五保供养标准稳步提高，住房公积金制度覆盖面持续扩大。开工建设保障性住房24490套，基本建成17510套，改造农村危房39450户。千方百计加强民生建设。优先发展教育事业，2012年全市财政教育支出占公共财政支出的19.2%。职业教育成效显著。文化、体育、卫生、人口与计划生育、老龄、妇女儿童、残疾人、慈善、红十字等社会事业全面发展，人民群众的精神文化生活更加丰富。加强农村基础设施建设，新改建农村公路512公里，改造大中危桥33座、1780延米，新增农村沼气用户11337户，解决了37.8万农村人口和7.3万在校师生安全饮水问题。对136个村实施扶贫整村推进，又解决12.4万贫困人口脱贫。推进林业生态市建设，全年完成造林36.97万亩。加强淮河源生态功能区保护和饮用水水源地保护，南湾湖饮用水水源地污染源搬迁工作取得阶段性成果。大力开展环境综合整治，全市人居环境得到持续巩固提升。全面实施质量立市战略，严厉打击侵犯知识产权和制售假冒伪劣商品行为，黄国粮业获得省长质量奖，潢绣防水建材、文新茶叶、宏润冷冻获得市长质量奖。积极创新社会管理，平安信阳建设扎实推进，社会治安综合治理得到加强，公共安全应急管理体系不断完善，食品药品、检验检疫和安全生产监管力度不断加大，信访形势持续平稳，社会大局和谐稳定。

　　（六）坚持“两转两提”，行政效能持续提高。持续深化“两转两提”，积极开展“创先争优”，严格落实党风廉政建设责任制，加强惩治和预防腐败体系建设，政府自身建设迈出新的步伐。坚持依法行政，自觉接受人大及其常委会的法律监督、工作监督和政协民主监督，认真办理人大代表建议和政协提案，强化行政监察和审计监督。深化行政审批制度改革，积极推进公共资源交易平台和行政效能电子监察平台建设，政府公共服务集成管理水平全面提升。政务公开深入推进，行政复议、行政执法监督工作继续加强。圆满完成居委会换届选举工作，城乡社区和基层自治组织服务功能进一步健全。严格执行“五禁止”、“十不准”规定，突出治理庸、懒、散、软问题，干部作风建设进一步加强，行政效能进一步提高，发展环境进一步优化。

　　加强社会主义核心价值体系学习教育和精神文明建设，公民道德素质和社会文明程度明显提升。积极开展“双拥”共建活动，军政军民团结进一步加强，民兵预备役建设取得新进展。驻信解放军和武警、消防部队在支持地方建设、维护社会稳定等方面作出了重要贡献，工会、共青团、妇联和科协、文联、社科联、工商联等人民团体在经济社会发展中发挥了不可替代的作用，民族、宗教、外事、侨务、对台工作全面加强，统计、物价、机关事务、地质、气象、地震、人防、档案、史志等工作取得新成绩。

　　各位代表！

　　在困难和挑战面前，全市经济社会发展取得这样的成绩来之不易。这是我们全面贯彻落实党的路线方针政策的结果，是省委、省政府和市委正确领导的结果，是人大、政协监督支持的结果，是全市人民共同奋斗的结果。在此，我代表市人民政府，向全市广大工人、农民、知识分子、干部职工，向驻信部队和武警官兵、公安干警以及社会各界人士致以崇高的敬意和衷心的感谢！

　　各位代表！

　　我们也清醒地看到，我市前进道路上还面临诸多困难和挑战：经济基础薄弱、经济总量不高、人均水平较低的基本市情尚未根本改变，加快发展的任务依然艰巨；工业化、城镇化水平不高，城乡、县区之间发展还不平衡，三次产业、三大需求之间结构仍不协调，加快发展的动力有待加强；科学发展的体制机制有待完善，基础支撑能力有待提升，土地、资金、人力等发展瓶颈依然存在；城乡居民收入整体不高，财政支撑和保障公共服务的能力仍然较弱，一些地方和领域涉及群众切身利益的就业、就学、就医、住房、安全生产等问题尚未得到很好解决，改善民生和加强社会管理的任务依然繁重；政府职能转变还有差距，一些政府工作人员的思想观念、工作方法、工作作风、能力素质与新形势新任务要求还不相适应，一些领域的不正之风还比较严重。对此，我们始终保持清醒忧患，继续采取有效举措，认真加以解决。

　　二、2013年政府工作的总体要求和主要目标

　　今年是深入贯彻落实党的十八大精神和实施《中原经济区规划》的开局之年，是全面完成“十二五”规划承前启后的关键一年，也是信阳勇当中原经济区建设前锋大有可为的一年，做好今年的政府工作意义重大。

　　当前，我市面临着难得的发展机遇。一是在党的十八大之后，国家将出台诸多加快发展的利好政策，加大对中西部地区和薄弱环节的扶持力度，支持革命老区和欠发达地区发展，信阳将享受到更多的政策支持和改革“红利”。二是《中原经济区规划》全面实施，信阳有30多项重点工作和重点项目列入规划，在构建沿京广发展轴、发展沿淮经济带、建设地区性交通枢纽和豫东南承接产业转移示范区，打造电子信息产业基地、绿色食品和新型建材产业集群、大别山生态红色旅游区等方面有了更大的发展平台。三是经过多年的发展积累，我市以“四通道一纽带”为支撑的前沿优势、以15个产业集聚区为主体的发展载体、以农村改革发展综合试验区为抓手的创新平台，正在聚合成为我市承接产业转移合作的新优势和正能量。同时，我市新型城镇化蕴含巨大的发展空间和潜力，未来几年大规模农村人口进入城市，必将产生极大的投资需求和消费需求。这些都为我市经济社会发展奠定了坚实基础，创造了良好条件。只要我们紧紧抓住并积极用好这些机遇，就一定会在魅力信阳建设的征程中迈出新的更大的步伐。

　　今年政府工作的总体要求是：深入贯彻落实党的十八大和中央、省委经济工作会议及市四次党代会精神，坚持以邓小平理论、“三个代表”重要思想、科学发展观为指导，紧紧围绕主题主线，坚持“四个重在”的实践要领，按照“持续求进，务实发展，积极作为”的总要求，全面落实《中原经济区规划》，牢牢把握“一市一区两枢纽一基地”的战略任务，以提高经济增长质量和效益为中心，以改革开放为动力，着力扩需求、稳增长、促转型、惠民生，在加快信息化进程中持续探索“两不三新”三化协调科学发展路子，努力实现经济社会持续健康较快发展。

　　今年全市经济社会发展的主要目标是：生产总值增长11%左右；地方公共财政预算收入增长13%；全社会固定资产投资增长22%；社会消费品零售总额增长16%；外贸进出口增长20%；实际利用外商直接投资增长20%；城镇居民人均可支配收入、农民人均纯收入实际增长9%以上，力争与经济增长同步；城镇新增就业9万人以上；人口自然增长率控制在6.5‰以内；居民消费价格涨幅3.5%左右；节能减排完成省下达的目标任务。

　　做好今年的政府工作，必须把握好以下原则：一是必须融入全局。主动融入全面建成小康社会和中原经济区建设全局，紧紧围绕魅力信阳建设的总体目标，遵循发展规律，拓展发展空间，增强发展能力，提升发展水平。二是必须乘势而为。按照党的十八大作出的“五位一体”的总布局，按照促进工业化、信息化、城镇化、农业现代化同步发展的新要求，按照省委、省政府坚持“总要求”、转变“立足点”、突出“四着力”、抓好“三重点”的总部署，凝心聚力，务实重干，科学运作，乘势而上。三是必须创造优势。既要认真研究优势、提升优势、发挥优势、放大优势，全力打好“前沿牌、生态牌、民智牌”，为经济社会发展提供支撑和保障，又要大力实施创新驱动战略，积极培育和创造全市发展的新活力、新动力、新体系、新优势。四是必须持续前行。要持续科学发展的意识、思路、举措和机制，不动摇、不懈怠、不折腾；要持续先行先试、敢于担当、善做善成的精神，转变观念做，转变方法做，转变作风做，一以贯之做，持之以恒谋发展；要持续推动改革、着力发展、保障民生、促进和谐，深谋远虑抓大事，千方百计解难事，雷厉风行办急事，一年一个台阶，一步一个脚印，扎扎实实前行，推动信阳在科学发展的道路上越走越好，力争到2020年提前实现全市生产总值和城乡居民收入比2012年翻一番，与全国、全省同步全面建成小康社会。

　　三、2013年的工作重点

　　今年，重点抓好八个方面的工作：

　　（一）持续强化招商引资，着力提升对外开放水平。全面落实对外开放基本省策，全方位拓宽开放领域、提升开放层次、扩大开放效果，打好开放招商工作总体战。持续深化“四一”招商。以更大力度推进“四一”招商，全市实际利用市外资金力争突破270亿元。重点依托产业集聚区，突出主导产业，围绕产业链条图谱，逐一制定专项行动方案，持续开展以商招商、延链补链招商和专业化、集群式招商，努力引进一批龙头型、基地型、集群型项目提高主导产业配套和竞争能力，加快形成特色产业集群。遵循产业转移规律，坚持开放引进外地企业与择优扶强本地企业并重、引进技术密集型产业与承接劳动密集型产业并重、引进龙头企业与吸引配套企业并重，通过大规模承接集群式、链条式产业转移，补齐缺失链条，补强薄弱环节，努力在承接产业转移中形成新的竞争优势。全面拓展开放领域。既要持续推进工业领域开放，也要高度重视和加快推进城乡建设、农业、服务业、社会事业等领域开放，落实好鼓励和引导社会资本进入社会事业的配套支持政策，努力吸引更多境内外资金进入高技术产业、战略新兴产业、现代服务业和现代农业，参与新型城镇化和农村基础设施建设。加强与国内外知名商业企业合作，引导其商业网点加快向我市战略布局。积极开展金融招商，吸引境内外金融、证券、保险等机构和各种基金、金融中介组织来我市设立分支机构，鼓励市外、境外资金参股本地法人金融机构。高质量办好第21届信阳国际茶文化节，进一步提升对外开放水平。完善配套服务体系。加快信阳海关建设，力争年内建成通关；积极谋划陆路口岸，加快筹建信阳保税物流中心，全面提升检验检疫水平，进一步完善对外开放基础条件。

　　（二）持续强化新型城镇化引领，着力加快城乡发展一体化。突出发挥新型城镇化的引领作用，推进产业集聚、人口集中、土地集约，探索走好符合信阳实际、具有信阳特色的新型城镇化道路。优化城市功能布局，提升中心城市辐射带动能力。以建设区域性中心城市为目标，加快修编《信阳市城市总体规划（2013-2030）》，进一步扩大中心城区控制性详规覆盖面。着力构建"6+3"中心城市框架，进一步加快老城改造和新区开发步伐，推进商务中心区和特色商业区建设，不断增强羊山行政中心商务区和浉河区、平桥区综合承载能力，提升信阳高新技术产业开发区、上天梯产业集聚区和南湾湖风景区建设水平。继续推进罗山县城、鸡公山文化旅游综合开发试验区、明港镇与信阳中心城区组团式发展，加快组团间的快速通道、产业配套和公共服务体系建设，进一步提升组团服务功能和吸纳就业能力。加快中心城区路网和高铁站片区建设，认真完成浉河三期年度治理任务，推进新七大道贯通工程，规划建设国际家居产业小镇路网和城市内外环线，积极谋划环城高速，着力打通城市断头路和出入口道路，提升中心城市综合辐射带动能力。增强城镇综合承载能力，有序推进农业转移人口市民化。大力实施“一基本两牵动”，推动以产兴城、产城融合，解决好进城农民就业问题；进一步优化中小学校布局，加强县城、小城镇、城乡结合部和产业集聚区周边学校建设，着力解决进城务工人员随迁子女入学问题；认真落实国家房地产调控政策，增加保障性住房和普通商品住房供应，把具备条件的农业转移人口纳入城镇住房保障体系，促进房地产业健康发展；加快城镇综合交通、环保、水电气热供应等基础设施和文化、卫生等公共服务设施建设，持续提升城镇综合承载能力。大力实施城市精细化、数字化管理，持续创建国家卫生城市，全面开展城市环境综合整治，优先发展城市公共交通，不断改善和提升人居环境。加快构建现代城镇体系，促进城乡一体化发展。继续优化城镇空间布局，统筹推进中心城市、县城、中心镇和新型农村社区协调发展。继续把县城作为推进城镇化的重点，提升规划建设标准，提高综合承载能力，鼓励引导就近城镇化。全面落实固始直管县政策，切实加快潢光一体化进程。充分发挥小城镇连接城乡的关键节点作用，积极创建绿色低碳小城镇，鼓励和支持有条件的小城镇扩大规模，稳步发展成为小城市。坚持把新型农村社区建设作为城乡一体化的切入点，按照省委、省政府要求，完善规划、优化布局，因地制宜、突出特色，示范带动、提升水平，积极稳妥推进新型农村社区建设。

　　（三）持续强化新型工业化主导，着力构建现代产业体系。坚持更多地关注工业、学习工业、研究工业、支持工业，加快构建现代产业体系，进一步提升新型工业化水平。加快产业集聚区建设。按照“四集一转”要求，进一步提升产业集聚区发展层次，努力实现“两提两扩两优”目标。进一步提升速度、提高水平。突出围绕主导产业发展，着力提高产业集聚区项目、工业项目和龙头型项目占全市固定资产投资的比重，加快企业集聚、产业集群速度，努力把信阳高新技术产业开发区争创为国家高新技术开发区，把潢川经济技术开发区争创为国家经济技术开发区。进一步扩大规模、扩大效果。力争全市产业集聚区完成年度投资750亿元，规模以上主营业务收入超过1100亿元，吸纳就业人员超过18万人，提高产业集聚区的产出率、就业率和贡献率。进一步优化配套、优化机制。坚持建设服务与生产服务并重、项目配套与生活配套并重、发展保障与社会保障并重，加快产业集聚区基础设施建设，抓紧完善现代物流、金融担保、职业培训、技术研发、检验检测等生产性服务功能，尽快配套社区服务、职工公寓、医疗保健、商业网点等生活性服务设施。认真落实招商项目择地落户机制、“四集中”制度、“零阻碍”服务、奖惩考核办法等一系列支持政策、配套措施和激励机制，持续增强产业集聚区发展后劲。加快产业转型升级。以市场需求为导向，以发展高端、终端和高附加值产品为重点，以承接产业转移为途径，大力发展电子信息、现代家居等高端制造和新型建材、绿色食品三大战略支撑产业，培育一批特色产业集聚区和专业园区；以精深加工、技术改造为重点，加快推动钢铁、造船、纺织等传统优势产业改造升级；以加快优势转化、核心技术研发为重点，积极培育生物医药、新能源等战略新兴产业，加快电动汽车的推广应用，支持生物医药企业实现转型升级。加快重大项目建设。以新上企业项目为增长点，以龙头企业改扩建为重点，以推动创新能力建设为着力点，大力实施产业集聚区重点项目攻坚计划，加快激蓝OLED、信阳国际家居产业小镇等140个投资1000万元以上的重大工业转型升级项目建设，确保完成年度投资150亿元，拉动工业经济快速增长。加快服务业发展。围绕构建区域性物流枢纽，抓好金牛物流产业集聚区、信阳交通枢纽物流园区和潢川铁路综合物流港等大型物流基础设施和重大物流项目建设，积极培育大型物流企业，发展壮大现代物流业；加强旅游规划管理，推进旅游资源整合，完善配套服务设施，提升旅游产业发展层次，力争全年接待游客突破1700万人次，实现旅游综合收入80亿元；加快推进一批定位明确、各具特色、优势互补、竞相发展的示范性文化产业园区建设，鼓励各类文化企业发展壮大。

　　（四）持续强化新型农业现代化基础，着力巩固“三农”工作好形势。认真贯彻落实今年中央一号文件要求和中央农村工作会议精神，切实把“三农”工作作为重中之重来抓，保供增收惠民生，改革创新添活力，不断强化农业基础地位。抓好稳定增产。落实政策，统筹规划，整合资金，集中投入，大力实施高标准粮田“百千万”建设工程，扎实推进粮食生产核心区建设和粮食高产创建工程，统筹推进水利、农机、科技、林业、交通、电力等综合配套设施建设，确保粮食种植面积稳定在1250万亩左右，粮食产量稳定在110亿斤以上。抓好集群发展。围绕提高农业综合效益，加快实施农业产业化集群培育工程，大力发展粮油、茶叶、油茶、花卉、畜禽、水产等特色农业，加快建设一批无公害、绿色、有机和地理标志认证农产品基地。依托农业产业化龙头企业，通过龙头带动、专业合作、协作配套、精深加工、品牌打造，着力提升信阳茶叶、潢川花木、罗山现代园林、潢川华英、光山羽绒、三高农牧、平桥现代牧业、山信粮油、黄国粮业、息淮弱筋小麦加工等一批现代农业产业化集群发展水平。大力实施主食产业化工程，推进“放心粮油”工程和“菜篮子工程”建设。抓好配套支撑。加强农业科技支撑、质量安全、防灾减灾等公共服务体系建设，积极发展农业保险，扩大基层农业技术推广体系、动植物防疫体系、农产品质量检测体系覆盖面，加快推进主要农作物生产全程机械化，建设和改造一批区域性农产品批发交易市场。积极推进农业经营体制机制创新，综合运用政策引导、项目扶持、金融服务、技术支撑、奖励补助等手段，加快发展新型农民合作组织，培育专业大户、家庭农场、龙头企业、农业标准化示范基地等新型农业经营主体。抓好改革试验。围绕国家关注、群众关切的重大问题，积极发挥先行先试优势，进一步深化农村改革发展综合试验区建设，不断在农村土地流转、金融创新、专业合作、社会保障、城乡统筹、现代农业产业化、基层组织建设等方面探索新经验，逐步形成一系列农业持续发展、农民稳定增收、农村持久繁荣的制度体系，努力为全省、全国农村改革发展作出新贡献。抓好政策落实。严格落实强农惠农富农政策，多渠道增加农民收入。积极引导金融机构加大涉农信贷投放，确保涉农信贷增量占比不低于上年。紧紧抓住大别山片区扶贫攻坚机遇，认真落实行业扶贫、专业扶贫、社会扶贫等相关政策，力争实现12万农村贫困人口脱贫。

　　（五）持续强化基础设施建设，着力增强支撑保障能力。坚持以项目建设为抓手，进一步加快交通、水利、能源、信息等基础设施重大项目建设，为可持续发展提供支撑保障能力。加快构建现代综合交通体系。围绕构建综合交通和现代物流枢纽，认真编制好规划、储备好项目、组织好建设，加快形成铁路、公路、航空、水运等各种交通方式高效衔接、适度超前、功能配套的现代综合交通运输体系，力争完成年度投资50亿元以上。加快推进宁西铁路增建二线电气化改造和京港澳高速公路改扩建信阳段工程建设，力争信阳火车站站房改扩建工程同步实施。加快推进明港机场筹建工作，力争年内取得重大突破。加快省际公路和国省干线公路改造升级，年内完成干线公路改建150公里。结合村镇规划调整和新型农村社区建设，持续提高农村公路通达水平，配套改造大中桥2000延米。加快推进淮河淮滨港和淮滨至息县段航运建设工程，改造升级固始三河尖望岗码头，全面提高淮河航运能力。加快水利工程建设。加大出山店水库前期工作力度，力争年内取得重大进展。大力推进淮干一般堤防加固及史灌河治理工程前期工作，加快建设南湾、鲇鱼山、梅山灌区续建配套与节水改造工程，继续实施水土保持综合治理、小型病险水库除险加固和中小河流治理工程，开工建设大中型病险水闸除险加固工程，全面提高抗灾减灾能力。继续推进农村居民和师生饮水安全工程建设，争取再解决30万农村居民饮水不安全问题。加快能源保障体系建设。大力创建绿色能源示范县，进一步优化能源结构和布局，加快可再生能源开发利用步伐。建成驻马店-信阳成品油管道及配套下载库工程，加快建设南阳-信阳天然气管道，谋划天然气液化和储备库项目，提高油气保障能力。加快信阳东500千伏输变电工程及配套电网建设，积极实施新一轮农网改造升级工程，着力破解电网供电瓶颈，确保信阳电网供电能力提高一倍。加快信息基础设施建设。充分发挥信息化在“三化”协调发展中的带动作用和提升作用，逐步实现与“三化”融合发展、同步发展。加快推进中国移动河南TD工程、中国电信3G网络工程、中国联通智慧中原工程等在信阳实施的重大项目，大力发展呼叫产业，实现全市3G网络城乡全覆盖和信息网络宽带化升级。积极推进广播电视网络数字化、双向化和互联互通改造，力争年内完成市区有线电视数字化整体转换。

　　（六）持续强化改革创新，着力释放经济社会发展活力。把改革作为最大的“红利”，用深化改革来推动经济快速发展，推动经济发展方式转变，推动要素保障机制创新。积极推进重点领域改革。继续深化国有企业改革，加快市直机关所属企业脱钩改制步伐，全面推开厂办大集体企业改革，积极发展与央企及国内外重点企业的战略合作；进一步深化集体林权制度和县级国有粮食企业产权制度及供销体制改革，加快推进县供电代管体制改革。继续深化医药卫生体制改革、教育体制改革和文化体制改革，积极推进财税金融、价格、收入分配等领域改革。创新要素保障机制。强化用地保障。积极推进“人地挂钩”政策试点，大力实施土地储备制度，持续开展对囤地圈地、违规用地、土地闲置浪费等问题的专项治理，通过城乡建设用地增减挂钩、“三项整治”、盘活存量土地、集约节约用地等途径缓解用地压力。坚持有保有压原则，新增建设用地计划指标优先支持重点项目和重点载体建设。强化资金保障。完善融资机制，创新金融产品，拓宽融资渠道，深化银企、银区（产业集聚区）合作，提高国有“四资”运作水平。大力发展资本市场直接融资，积极推动“准市政债”发行和“区域集优债务”融资，加快上市企业再融资步伐。大力支持非公有制经济和民营企业、中小微企业发展，进一步完善土地、厂房等产权手续，提高企业自身融资和抵押能力。强化用工保障。大力实施全民技能振兴工程，注重把提高劳动者就业技能与解决企业用工问题等相结合，与落实公共租赁房政策相结合，积极探索有利于农村人口向城镇转移的体制机制，努力为部分农村劳动力全家进城、实现稳定就业创造条件。搭建科技创新平台。大力实施传统产业技术创新工程、战略性新兴产业科技成果转化工程，着力推动传统产业改造升级，引领战略性新兴产业发展壮大。积极开展产业集聚区创新平台建设，着力构建以企业为主体、市场为导向、产学研相结合的自主创新体系。大力实施籼改粳、OLED重大科技专项和自主创新专项，努力突破一批产业关键核心技术，培育一批拥有自主知识产权和“信阳制造”的品牌企业，增强产业核心竞争力。继续开展省长、市长质量奖和品牌产品创建活动，持续推进质量提升、标准引领和品牌带动工程。

　　（七）持续强化生态文明建设，着力培育区域竞争优势。切实把生态文明建设放在更加突出的位置，认真落实国家主体功能区规划，着力推进绿色发展、循环发展、低碳发展，增强可持续发展能力。加强资源能源集约节约利用。严格落实节能减排目标责任制，实行更加严格的环境准入制度和固定资产投资节能评估审查制度，增强企业和全社会节能减排内生动力，继续淘汰落后产能，严格控制能耗不合理增长。着力实施一批节能减排重大项目和示范工程，推动资源能源利用方式根本转变，形成集约节约的生产生活方式，完成节能减排目标。加强资源综合利用，大力发展节能环保产业，推进固始县、潢川县产业集聚区、华英集团等省级循环经济试点建设，着力提升循环经济发展水平。加强生态环境保护。进一步加大对重点流域、重点区域、重点行业污染综合整治，加快推进垃圾处理、污水处理、中水回用、污泥处置、雨污分流等工程建设，加强对水、大气、土壤、重金属、汽车尾气、畜禽养殖污染等检测和治理。严格贯彻实施《河南省信阳南湾水库饮用水水源保护条例》，着力加强饮用水水源地保护，持续推进淮河源国家级生态功能保护区建设和南湾湖国家重点湖泊环境治理。积极开展农村环境连片综合整治，控制农村面源污染。严格落实主要污染物排放总量预算管理制度和全流域生态补偿制度，积极探索实施排污权有偿使用和交易。加强生态系统建设。积极创建全国绿化模范城市，大力实施林业生态市建设提升工程，全年计划完成造林任务33万亩。继续推进天然林保护、水源涵养地保护、湿地保护和山区生态体系、农田防护林体系、生态廊道网络建设、村镇绿化等重点林业生态工程建设，开工建设一批重大生态修复工程，严厉打击乱砍滥伐森林行为，积极构建淮河中上游和大别山区重要生态屏障，努力建设生态信阳、美丽信阳。

　　（八）持续强化社会事业发展，着力加强和谐社会建设。持续做好民生工作，解决好人民群众最关心最直接最现实的利益问题。努力实现更高质量的就业。实施更加积极的就业政策，把承接产业转移、加快服务业发展与促进农村人口转移结合起来，多渠道开发就业岗位，重点解决好高校毕业生、农村转移劳动力、城镇困难人员、退役军人和残疾人就业，扩大就业人数，提高就业质量。进一步消除进城和跨地区就业的制度性障碍，大力扶持农民工回乡创业，带动更多的新型农村社区居民和农村劳动力就近就地实现转移就业。努力做好更大范围的社会保障。坚持全覆盖、保基本、多层次、可持续方针，以增强公平性、适应流动性、保证可持续性为重点，加快完善覆盖城乡的社会保障体系。积极推动城镇职工养老保险与城乡居民养老保险衔接，城镇职工医保与居民医保、新农合衔接，大病救助与临时救助衔接，尽快实现各类参保人员在地区之间、制度之间的顺畅转移和衔接。强化政策引导，将各类人员纳入相应社会保险覆盖范围，稳步提高社会保障水平。大力发展老龄、残疾人、慈善、红十字事业，建立健全以居家养老为基础、社区服务为依托、机构养老为支撑的社会养老服务体系，加快残疾人康复、托养中心建设。进一步扩大住房公积金制度覆盖面。继续推进保障安居工程建设，全年新开工建设保障性住房2.25万套。努力办好人民满意的教育。加快推进学前教育三年行动计划，不断提升学前教育质量。积极推进义务教育薄弱学校改造，促进义务教育均衡发展。实施一批普通高中改造工程，不断改善办学条件。大力发展高等教育，加快推进市职教园区建设步伐。积极推广平桥职教园区“学校+企业+实训基地”模式和新县涉外劳务培训模式，打造信阳职业教育品牌，努力实现就学与就业一体，招工与招商同步。进一步加大投入、落实制度、强化责任、加强监管，确保校园和学生安全。努力发展群众喜爱的文化。加大对信阳历史文化的研究和成果运用，继续总结、挖掘和弘扬大别山精神，支持各县区以特色文化引领县域经济发展，推动文化大发展大繁荣。继续推进广播电视村村通、文化信息资源共享、农村数字化电影放映、文化下乡、流动舞台配送等重点文化惠民工程，不断提升公共文化设施服务能力。稳步推进文化遗产保护传承，建立健全代表性传承人保护政策。不断完善公共体育设施，积极开展全民健身运动，办好第五届全国公路自行车公开赛和我市第四届全民运动会。努力提升医疗卫生服务能力。加快推进市级综合医院、专科医院和全科医生临床培训基地建设，继续实施县级医院、重大疾病防控、乡镇医院、农村120急救指挥中心、妇幼保健等建设项目，完善重大疾病防控、卫生监督、妇幼保健、农村应急救治等公共卫生服务网络，健全基层医疗卫生服务体系，提升基层医疗卫生服务能力。全面提高出生人口素质，持续稳定低生育水平。努力加强和创新社会管理。坚持依法办事和依法维权相结合，不断完善社会稳定风险评估机制，提高社会管理科学化水平。积极完善和推广“四家”工作法，畅通信访渠道，规范信访秩序，妥善处理各类矛盾和问题，进一步密切党同人民群众的血肉联系。持续开展“打非治违”活动，深化重点行业和领域的专项整治，坚决遏制重特大事故发生。切实加强食品安全风险监测体系建设，确保食品安全。深入开展打击侵犯知识产权和制售假冒伪劣商品专项整治行动，维护公平竞争的市场秩序。持续深化平安信阳建设，加大社会治安综合治理，加强法律服务和援助，依法打击各类违法犯罪活动，保持社会大局和谐稳定。

　　加强国防教育，增强全民国防意识，抓好民兵预备役和人民防空工作，积极支持国防建设和驻信解放军、武警、消防部队建设；认真落实优抚安置政策，继续以军地互办实事为抓手，深入开展“双拥”共建活动，巩固和发展军政军民团结。大力支持工会、共青团、妇联和科协、文联、社科联、工商联等人民团体发挥桥梁纽带作用，全面落实民族、宗教政策，切实做好外事、侨务、对台工作。认真做好第三次全国经济普查。继续抓好机关事务、地质、气象、地震、档案、史志等各项工作。

　　今年，在完成省定“十项重点民生工程”的同时，继续抓好我市“双十”工程。2013年“双十”工程安排意见已经印发，请予审议。

　　四、切实提高政府工作水平

　　深入学习贯彻党的十八大精神，认真落实中央关于改进工作作风、密切联系群众的八项规定，坚持用领导方式转变加快发展方式转变，深入推进“两转两提”，建设务实政府，树立务实形象，全面提升政府工作水平。

　　（一）转变政府职能。正确处理政府和市场的关系，推进政企、政资、政事、政社分开，发挥市场、企业、社会组织和中介机构作用，减少对微观经济活动干预，推动政府职能向创造良好发展环境、提供优质公共服务、维护社会公平正义转变。严格落实“五禁止”、“十不准”规定，继续清理、精简和规范行政审批事项，简化办事程序，提高工作效率和办理质量。

　　（二）严格依法行政。深入开展“六五”普法工作，不断强化社会主义法治理念，增强全社会学法尊法守法用法意识。严格按照法定权限和程序行使权力、履行职责、规范行为，自觉用法治思维和方式调节经济运行和管理社会事务。认真落实行政执法责任制和过错责任追究制，完善行政执法监督制约机制，严格规范公正文明执法。严格按政府工作规则议事，坚持把公众参与、专家论证、风险评估、合法审查和集体讨论决定作为重大决策的必经程序，依法进行科学民主决策。做好行政调解等工作，办好行政复议案件。加强政务公开和政府信息公开，健全完善政府工作运行机制，推进公共权力公开透明运行，确保行政权力依法规范行使。

　　（三）切实改进作风。认真贯彻执行中央和省委、市委关于改进工作作风、密切联系群众的各项规定，认真开展以为民务实清廉为主要内容的群众路线教育实践活动，讲实话、干实事、求实效，以踏石留印、抓铁有痕的劲头把各项工作做得更实。切实改进工作方法，坚持“三具两基一抓手”，多做打基础、管长远、积后劲的工作，多办顺民意、解民忧、惠民生的实事，严禁损害群众利益。切实改进调研方式，轻车简从、简化接待，多到情况复杂、矛盾困难集中、群众意见较多的地方，帮助解决问题。切实改进文风会风，减少检查评比，强化行政问责，力戒形式主义、官僚主义，真正把精力用在谋发展、抓落实上。切实改进考评机制，坚持在重大决策的执行、重点工作的推进、重大项目的实施中考核干部、评价干部、使用干部。

　　（四）坚持廉洁从政。认真贯彻中纪委二次全会和省、市纪委三次全会精神，全面落实党风廉政建设责任制，严格执行廉洁从政各项规定，进一步完善惩治和预防腐败体系，严肃查处违法违纪案件。加大纠风和专项治理力度，强化对财政资金、重大投资项目、土地招拍挂、政府采购、国有资产资源经营管理等重点领域的行政监察和审计监督，加强对领导干部经济责任审计，重点纠正公共服务行业和教育、医疗、涉农、征地拆迁、涉法涉诉等领域损害群众利益的不正之风和突出问题，真正做到用制度管权、管财、管事、管人。大兴艰苦奋斗之风，厉行勤俭节约，反对铺张浪费，严控行政经费等一般性支出，严控楼堂馆所建设。大力加强学习型、服务型、创新型、廉洁型机关建设，健全“创先争优”长效机制，建设高素质公务员队伍。

　　各位代表！

　　作为人民的政府，我们将在市委的正确领导下，严格执行市人大及其常委会的决议、决定，自觉接受人大及其常委会的法律监督、工作监督和人民政协的民主监督，认真办理人大代表建议和政协提案，广泛听取民主党派、无党派人士和人民团体意见，主动接受社会监督和舆论监督，忠诚履职，尽心尽力，更好地为人民服务，对人民负责，受人民监督，让人民满意。

　　各位代表！

　　党的十八大为我们指明了前进的方向，中原经济区建设为我们带来了新的重大机遇，魅力信阳建设迈入了新的发展阶段。面对发展的新形势，面对人民的新期待，我们深感责任重大，使命光荣！让我们紧密团结在以习近平同志为总书记的党中央周围，持续求进，务实发展，积极作为，勇当中原经济区建设前锋，确保完成今年各项目标任务，不断开创魅力信阳建设的新局面！
